# A Bayesian System to Detect and Track Outbreaks of Influenza-Like Illnesses Including Novel Diseases: Algorithm Development and Validation

**DOI:** 10.2196/57349

**Published:** 2024-08-13

**Authors:** John M Aronis, Ye Ye, Jessi Espino, Harry Hochheiser, Marian G Michaels, Gregory F Cooper

**Affiliations:** 1 Department of Biomedical Informatics University of Pittsburgh Pittsburgh, PA United States; 2 Department of Pediatrics University of Pittsburgh School of Medicine UPMC Children's Hospital of Pittsburgh Pittsburgh, PA United States

**Keywords:** biosurveillance, outbreak, novel disease, natural language processing, disease modeling, Bayesian modeling, influenza, influenza-like illnesses, novel diseases, public health, COVID-19, SARS-CoV-2, coronavirus, hospital, hospitals, emergency department, patient care, NLP, algorithm, respiratory syncytial, human metapneumovirus, parainfluenza, Pennsylvania, enterovirus D68, surveillance

## Abstract

**Background:**

The early identification of outbreaks of both known and novel influenza-like illnesses (ILIs) is an important public health problem.

**Objective:**

This study aimed to describe the design and testing of a tool that detects and tracks outbreaks of both known and novel ILIs, such as the SARS-CoV-2 worldwide pandemic, accurately and early.

**Methods:**

This paper describes the ILI Tracker algorithm that first models the daily occurrence of a set of known ILIs in hospital emergency departments in a monitored region using findings extracted from patient care reports using natural language processing. We then show how the algorithm can be extended to detect and track the presence of an unmodeled disease that may represent a novel disease outbreak.

**Results:**

We include results based on modeling diseases like influenza, respiratory syncytial virus, human metapneumovirus, and parainfluenza for 5 emergency departments in Allegheny County, Pennsylvania, from June 1, 2014, to May 31, 2015. We also include the results of detecting the outbreak of an unmodeled disease, which in retrospect was very likely an outbreak of the enterovirus D68 (EV-D68).

**Conclusions:**

The results reported in this paper provide support that ILI Tracker was able to track well the incidence of 4 modeled influenza-like diseases over a 1-year period, relative to laboratory-confirmed cases, and it was computationally efficient in doing so. The system was also able to detect a likely novel outbreak of EV-D68 early in an outbreak that occurred in Allegheny County in 2014 as well as clinically characterize that outbreak disease accurately.

## Introduction

### Background

Respiratory viruses are responsible for annual and oftentimes overlapping outbreaks in human populations. This overlapping disease activity confounds the diagnosis and treatment of patients presenting with influenza-like illnesses (ILIs) and the associated high caseloads stress the clinical and logistical capacity of the health care system. Thus, accurately detecting and tracking overlapping outbreaks due to these viruses are important tasks with public health implications and clinical repercussions for those at high risk [1-5]. The ideal surveillance system will notice an outbreak after just a few cases that may be distributed across several shifts at multiple hospitals. An individual physician may see just 1 or 2 cases, which might seem inconsequential to them and not worthy of mention, but an automated surveillance system, such as we describe here, can gain statistical power and timeliness by aggregating data across an entire region. The proposed system can harness the entire set of patients and their symptoms who present to all the emergency departments (EDs) in a region. By harnessing the sheer volume of this information, it may recognize cases and patterns that would elude human observers early in the outbreak, and thus, serve as a sentinel to detect and characterize outbreaks early.

In addition to detecting and tracking known viruses of concern, the world is also faced with the emergence of novel viruses (or the re-emergence of previously quiescent viruses) and the diseases that they cause, as evidenced by the appearance of the SARS-CoV-2 worldwide pandemic. Early detection and tracking of a novel or re-emerging outbreak disease can be critical in informing both the care of individual patients and the decisions made by public health officials. While we hope to someday prevent the emergence of pathological viruses before they strike the human population [6], a more realistic goal for the near term is early detection and tracking [7]. Modeling known diseases that we expect to see provides a useful background against which to detect the emergence of new diseases that have a different clinical or epidemiological presentation.

### Previous Work

The most promising route to the early detection of outbreaks of pathological viruses is real-time surveillance of human and animal populations [2]. There are myriad approaches to the problem of disease surveillance based on different data sources and technologies. Most recent work has been based on data from laboratories [8] and sentinel physicians [9]. Social media, including Google Flu Trends, have been proposed as a useful tool [10], but initial efforts have not worked as well as expected [11-14]. More recent approaches to the use of social media are promising [15-18]. A variety of other sources, such as sales of over-the-counter medications, absenteeism, and traffic patterns have also been proposed [19].

Most of these data sources have significant limitations. For instance, laboratory tests of infectious pathogens can identify outbreak diseases that are known and tested for, but such tests are blind to emerging pathogens. Furthermore, laboratory tests are not always routinely performed or reported, especially in resource-challenged health care settings. Sentinel physicians and various “drop-in” surveillance methods have poor coverage, and individual physicians might not notice or appreciate isolated cases [20]. Methods based on data from social media [21], absenteeism [22], traffic patterns [23], etc, are nonspecific [24]. More importantly, these methods will only recognize anomalies after a substantial number of people have been affected, losing important time when an infectious disease could have been identified.

Syndromic surveillance [25] seeks to identify clusters of signs and symptoms among patients recorded during routine medical care, especially in hospital EDs. This approach has the advantages of both broad coverage (nearly every community in the United States is served by some ED) and the use of clinical information (including chief complaints, vital signs, clinical findings, etc). Unlike traditional systems that rely on voluntary reports from health care providers (who work with individual cases), syndromic surveillance systems use data about entire populations that are continuously and automatically acquired. Syndromic surveillance attains much of its timelines from the identification of syndromes before confirmed diagnoses are made.

To detect outbreaks of rare or novel threats, however, we must go beyond the traditional syndromic surveillance systems that only detect known syndromes, such as the Centers for Disease Control and Prevention (CDC) National Syndromic Surveillance Program [26]. To identify novel threats to public health, syndromic surveillance systems need to identify clusters of patients even if they are not characterized by a predefined syndromic grouping. The North Carolina Disease Event Tracking and Epidemiologic Collection Tool system [27] identifies clusters of related patients based on time of arrival to identify clusters of related ED visits in 30- and 60-minute windows. The study by Burkom et al [28] used a Fisher exact test to identify anomalous clinical terms in an 8-hour block of current chief complaints compared with a 30-day sliding baseline. Sets of anomalous terms are then presented to a human monitor for further investigation. The multidimensional semantic scan system [29] uses latent Dirichlet allocation to learn a set of syndromes directly from ED chief complaints. The learned syndromes include 25 “static” topics that correspond to common health conditions and a set of 25 “emerging” topics from recent data that may indicate newly emerging threats. The multidimensional semantic scan system also uses practitioner feedback to distinguish between relevant and irrelevant clusters. The system was extensively tested on data from New York City. In previous work [30], we described a system that builds a probabilistic model of normal (baseline) ILI activity using a large set of patient findings extracted from patient care reports with natural language processing. It then looks for statistically significant deviations from baseline normal activity. This system does not rely on just a small set of findings as might be extracted from patients’ chief complaints.

The ILI Tracker algorithm introduced here differs from previous work in several important ways. First, it uses a large set of findings extracted from patient care reports, not just chief complaints. This is important because most ILIs cannot be distinguished solely based on chief complaints but require a complete assessment to be recognized. This is especially important for the recognition of novel or emergent diseases that may only be detected by the presence of uncommon or unusual combinations of findings. Second, ILI Tracker explicitly models and tracks known ILIs. This provides a backdrop against which novel diseases can be detected even in the presence of modeled ILIs. Finally, because the ILI Tracker explicitly models known diseases and their symptoms, it can identify and characterize patients who do not fit the profile of the expected modeled ILIs and bring them to the attention of clinicians for further evaluation.

### Current Work

This paper describes the ILI Tracker algorithm that tracks the daily occurrence of a set of modeled ILIs in hospital EDs in a monitored region using natural language processing on patient care reports. A set of clinical findings is extracted from full-text patient care reports that are available at the time of care or shortly thereafter. These findings are used by machine learning algorithms to learn probabilistic models of a set of diseases. The models are used to determine the likelihood of each disease for each patient [31]. These likelihoods are then used to compute the expected prevalence of each disease in the EDs on each day. ILI Tracker also analyzes whether recent patient cases in the EDs are not well explained by the known diseases that it models. If so, it suggests the possible presence of a novel outbreak of a disease in the population.

The remainder of this paper first describes the ILI Tracker algorithm in detail. We build models for diseases such as influenza, respiratory syncytial virus (RSV), human metapneumovirus (hMPV), and parainfluenza (PIV) using data from 5 EDs in Allegheny County, Pennsylvania, from June 1, 2010, to May 31, 2014. We then present initial experiments on how well it can track those diseases from June 1, 2014, to May 31, 2015. Finally, we present a preliminary investigation of an algorithm based on the ILI Tracker for detecting the presence of an unmodeled disease.

## Methods

### The Algorithm

To diagnose a patient, a clinician must consider that patient’s findings as well as the prevalence of various diseases in the community. For instance, given a patient with a fever and cough, a high rate of influenza in the population will elevate the probability that the patient has influenza, whereas a high rate of SARS-CoV-2 will elevate the probability of SARS-CoV-2. The situation becomes more complicated when multiple viruses (with similar or overlapping symptoms) are circulating in the environment for which the rate of each must be accounted. Bayesian inference provides a principled way to do this.

We assume each patient has exactly one of the diseases {*dx_0_, dx_1_, …, dx_n_*}. If *Pr*(*dx_i_* | *findings*) is the probability that a patient has disease *dx_i_* (for some *i* in *0,…,n*) given their findings, and *Pr*(*dx_i_*) is the prevalence of that disease in the population at that time, then:



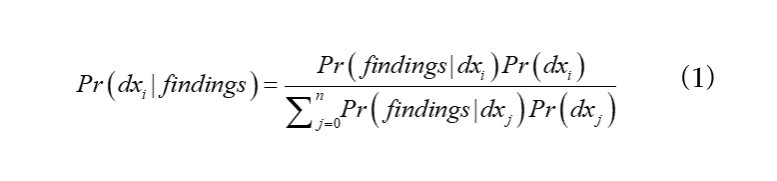



The denominator of Equation 1 is a sum over all the diseases we are modeling and is a normalizing factor, so we have:



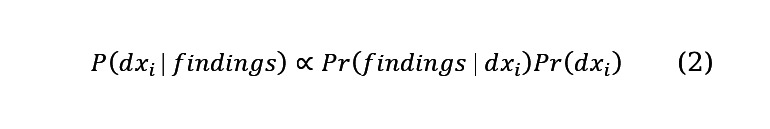



That is, given a patient’s findings, the probability that the patient has disease *dx_i_* is proportional to the product of the likelihood of their findings given *dx_i_* times the prior probability of *dx_i_*.

Using this formulation, we can compute the probability of each disease for each patient. The expected number of patients with each disease is the sum over the probability each patient has that disease. For example, suppose there are 50 patients and 20 have a 0.1 probability of influenza, while 30 have a 0.2 probability. Then the expected number of patients with influenza is 20×0.1+30×0.2=8. Given the expected number with each disease, we can compute the expected proportion of each disease. In the example above, the expected proportion of patients with influenza would be 8/50=0.16.

The ILI Tracker algorithm combines the above steps to compute the expected proportion of each disease each day. It starts with prior probabilities for each disease, computes the expected number of patients with each disease as above, and then uses the proportion of each disease as the prior probability of each disease on the next day. This process continues day by day. The remainder of this section provides the technical details of this algorithm.

We first introduce some notation. Let *days* be the sequence of days under consideration, *pts*(*d*) be the number of patients who visited the EDs on day *d*, *D*(*p,d*) be the set of findings (“data”) for patient *p* on day *d*, and 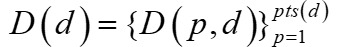 be all of the data for the patients on day *d*. Note that we number days starting with zero. We assume there is a set of modeled diseases *Dx*={*dx*_0_, *dx*_1_, …, *dx_n_*} where {*dx*_1_, …, *dx_n_*} are the diseases of interest and *dx*_0_ denotes other known diseases. Here, other represents a large set of known diseases including trauma, cardiac events, diabetic emergencies, etc, that can occur and are not one of the *n* modeled diseases.

For a patient *p* on day *d* with findings *D*(*p*,*d*), we can calculate the probability they have a particular disease *dx_i_*:



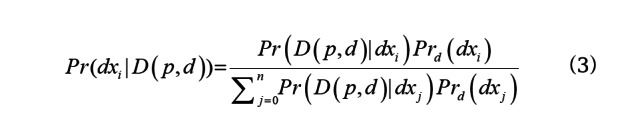



where *Pr_d_*(*dx_i_*) is the prior probability of *dx_i_* on day *d* and *Pr*(*D*(*p*,*d*)|*dx_i_*) is the likelihood of the patient *p*’s findings given they have disease *dx_i_*. We describe how we compute each of these quantities below. We compute the expected number of patients with each *dx_i_* on day *d* as:



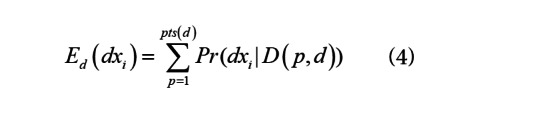



We can now estimate the posterior probability of each disease on day *d*:



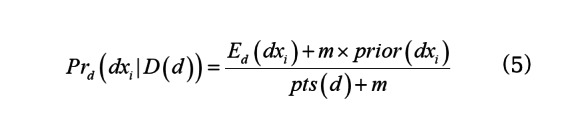



where *m* is the so-called equivalent sample size and *prior*(*dx_i_*) is the prior probability of disease *dx_i_*. The terms *m* and *prior*(*dx_i_*) in Equation 5 provide smoothing of the estimate that avoids relying too heavily on small values of *E_d_*(*dx_i_*) and *pts*(*d*) by augmenting the data with an additional *m* patients with diseases distributed according to *prior*(*dx_i_*). We specify *m* and *prior*(*dx_i_*) below. We then make the disease priors for day *d*+1 equal to the disease posteriors for day *d*.

In summary, the overall procedure is as follows. Set each prior probability *Pr*_0_(*dx_i_*) to initial values as described below. Then for each day *d*:

1. Compute *Pr*(*dx_i_* | *D*(*p*,*d*)) for each 
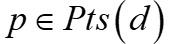
 using Equation 3.

2. Compute *E_d_*(*dx_i_*) for each *dx_i_* using Equation 4.

3. Compute *Pr_d_*(*dx_i_* | *D*(*d*)) for each *dx_i_* using Equation 5.

4. Set *Pr_d_*_+1_(*dx_i_*)=*Pr_d_*(*dx_i_* | *D*(*d*)) for each *dx_i_*.

Steps 1-4 are repeated for each successive day. That is, each day, *d* (beginning with the first day *d*=0), we start with a prior probability, *Pr_d_*(*dx_i_*), for each disease. We then use data from the patients in the EDs on day *d* to compute a posterior (updated) probability, *Pr_d_*(*dx_i_* | *D*(*d*)), for each disease. The posterior probability for each disease is then used as the prior probability for that disease, *Pr_d_*_+1_(*dx_i_*), the next day.

Our data spans the time from June 1, 2010, to May 31, 2015. We use the data from June 1, 2010, to May 31, 2014, to build and train disease models, and we start monitoring on June 1, 2014. The prior probabilities on June 1, 2014 for influenza, RSV, hMPV, and PIV were set to 0.033, 0.035, 0.005, and 0.003, respectively. We determined these values by running ILI Tracker on the data for March 1, 2014 to May 31, 2014 with priors on March 1, 2014 of 0.05, 0.05, 0.05, and 0.05, and using the resulting posterior probabilities from May 31, 2014 as the prior probabilities for June 1, 2014. The equivalent sample size, *m,* was set to 10.

### Detecting the Presence of Unmodeled Diseases

As mentioned, the better we can model the usual diseases we expect to see in the ED, the better we anticipate detecting novel diseases. The remainder of this section specifies how we do so.

We can regard the output of the ILI Tracker as a model of the types of patients who are in the ED each day. ILI Tracker assumes the presence of a fixed set of diseases that can be modeled using Bayesian networks with specified findings. If this assumption is satisfied then the model should explain the evidence (the patients and their findings) well and the probability of the data given by the model produced by ILI Tracker will be relatively high.

If any of these assumptions are violated, in particular, if there are patients with a novel, unmodeled disease, the probability of the data given by the model produced by ILI Tracker will likely be reduced compared with a previous period of time when only modeled diseases were present in the ED. If we track the probability of the data given by the output of ILI Tracker, a decrease in this daily probability may signal the presence of an unmodeled disease. An unmodeled disease may be a novel disease or a re-emergent disease that we are not currently modeling.

The day-to-day probabilities for each disease computed by ILI Tracker can be used to perform a posterior predictive check by computing the likelihood of the data each day given by the output of ILI Tracker as follows:



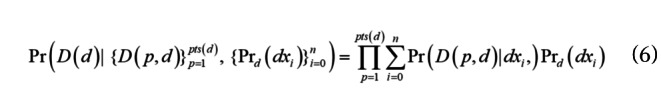



where 
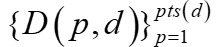
 are the findings on day *d* for each patient *p* and 
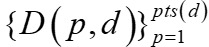
 is the set of prior probabilities of each disease on day *d* computed by ILI Tracker.

Let the null hypothesis be that the likelihood given by Equation 6 for the current day is the same as or greater than the likelihoods for all previously monitored days, up to 60 days. We compute a daily empirical P value, *p_d_*, for current day *d* as follows:

1. Compute 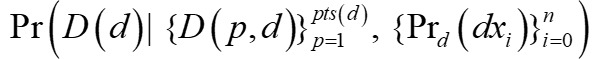.

2. Compute 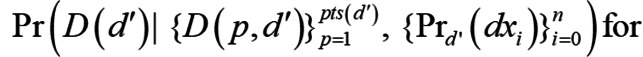



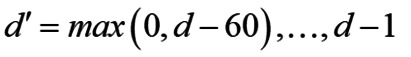
.

3. Set *p_d_* to the fraction of times the terms computed in step 2 are less than the terms computed in step 1.

That is, we compute the likelihood of the data for the previous (up to) 60 days, then compare the likelihood of the data on day *d* to those values. We say that day *d* is unusual if *P_d_*≤0.01. A sequence of unusual days, each with *P_d_*≤0.01, may signify an outbreak of a novel disease.

### Data and Modeling

Our data come from 5 University of Pittsburgh Medical Center (UPMC) hospitals serving Allegheny County in Southwestern Pennsylvania. As of the 2020 census, the population was approximately 1,223,000. Allegheny County encompasses the City of Pittsburgh which accounts for approximately 25% of the county population, with the remainder of the population being primarily suburban. The racial composition was approximately 75% White, 13% African American, and 12% other (including Native American, Asian, Pacific Islander, Hispanic, or Latino). The age distribution of the population is approximately 22% younger than the age of 18 years, 9% from 18 to 24 years, 28% from 25 to 44 years, 23% from 45 to 64 years, and 18% who were 65 years of age or older. UPMC serves approximately 60% of the ED visits in Allegheny County.

The training data set consisted of ED encounters at the 5 UPMC hospitals from June 1, 2010, to May 31, 2014, including 815 influenza, 414 RSV, 198 hMPV, 100 PIV that were laboratory-test positive, and 59,428 other visits. In particular, patient encounters with a positive laboratory test for influenza by polymerase chain reaction, direct fluorescent antibody, or viral culture were labeled as influenza. We use similar criteria for labeling patient cases with RSV, hMPV, and PIV. For training purposes, we excluded cases that have positive laboratory results for more than 1 virus. The 59,428 other visits were defined as visits in August 2010, 2011, 2012, or 2013, which did not have any influenza, RSV, hMPV, or PIV laboratory tests performed. These other cases were obtained from visits during August of those 4 years because relatively few outbreaks of these 4 diseases occurred during that month. The testing data set consisted of ED encounters from June 1, 2014, to May 31, 2015.

Free-text patient care reports were used as the input to the Topaz parser [32], which for each report generates the status (value) for each of the 79 clinical findings that clinical experts have deemed relevant to ILIs. The highest measured temperature was classified into 4 categories, which included ≥104.0 F (40.0 C), 100.4-103.9 F (38.0-39.9 C), <100.4 F (38.0 C), and unknown. Each of the remaining findings took the values present, absent, or unknown. The designation of “absent” indicated that the clinician had reported the finding as being absent (eg, “patient denies sore throat”). We discarded those findings with information gain scores (regarding disease diagnosis) of zero. Because there is a testing bias for ILIs across age groups, the age finding was not included.

**Figure 1:**
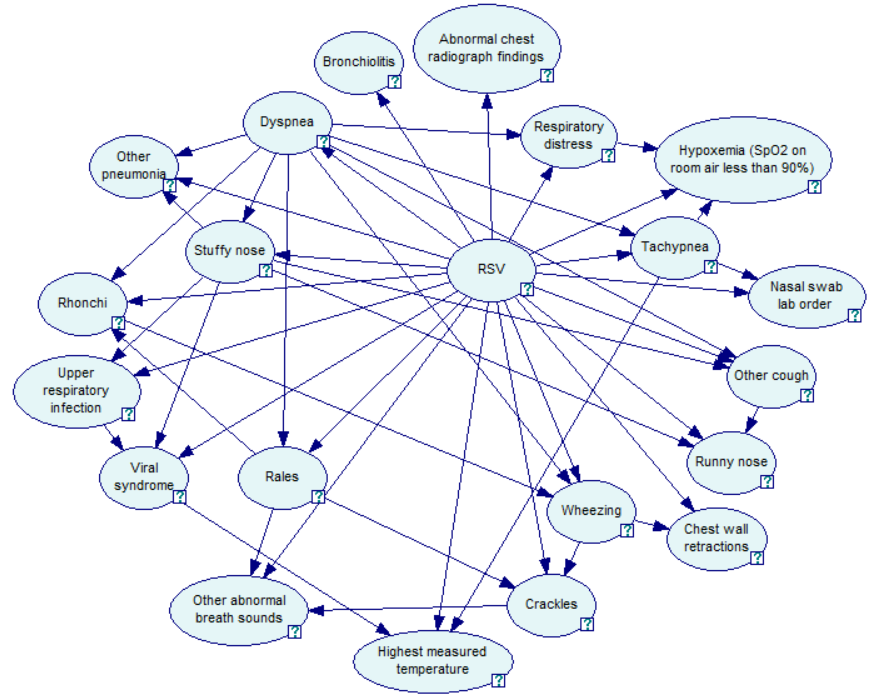
A Bayesian network model for the 20 most informative findings in the RSV model. RSV: respiratory syncytial virus; SpO2: measure of the saturation of peripheral blood oxygen.

Our approach to outbreak detection is based on modeling and tracking patients with known diseases and noting anomalies. This requires modeling each patient. To that end, we developed 5 Bayesian network disease models (ie, influenza, RSV, hMPV, PIV, and other) using the same search process to find each Bayesian network structure, as follows. We started with a naïve Bayes network structure in which all findings have an arc from the disease node to each finding node. We then used the K2 learning algorithm [33] to identify additional arcs among the clinical feature nodes which were assigned an arbitrary ordering. The search was based on the K2 Bayesian score with a restriction that each finding could have at most 2 parents beyond the disease node. After finding a network structure, we estimated from the data conditional probabilities in the network model. For instance, Figure 1 shows the relationships among the top 20 features of the RSV model [34].

We used the area under the receiver operating characteristic curve (AUC) [35] as the measure of discrimination performance. The AUCs obtained for influenza, RSV, hMPV, PIV, and other were 0.88, 0.92, 0.91, 0.89, and 0.90, respectively. In testing the performance of the influenza model, the disease-positive group consists of patient cases that have positive laboratory results for influenza. The negative test group consists of cases that either have negative laboratory results for influenza or have not had any laboratory tests performed for influenza. It is likely, however, that some of the patient cases without any laboratory tests for influenza will have influenza, which we would expect to have reduced the AUC that we report. An analogous situation exists for testing the RSV, hMPV, and PIV models.

When testing the model of other disease, the negative test group includes cases that have at least 1 positive laboratory test result for influenza, RSV, hMPV, or PIV. The positive test group consists of cases that either have negative laboratory results for all 4 respiratory diseases or have not had any of those tests performed. It is likely, however, that some of the cases without any tests performed will have one or more of the 4 respiratory diseases. Given this consideration and the discussion in the previous paragraph, the reported AUCs are likely to represent lower bounds on performance that would be obtained if the test case labels were more accurate.

### Ethical Considerations

The research protocol was approved by the University of Pittsburgh Institutional Review Board (study number 20030193). As no patients were enrolled in this study and no compensation was offered, we obtained a waiver of consent from the institutional review board. A UPMC-approved honest broker deidentified the data to meet the requirements of a limited data set before distribution.

## Results

This section reports the results we obtained in applying ILI Tracker to the data described above to estimate the presence of the outbreak diseases we modeled over time and to monitor for the presence of a novel disease in the population.

### Tracking of Known Diseases

**Figure 2:**
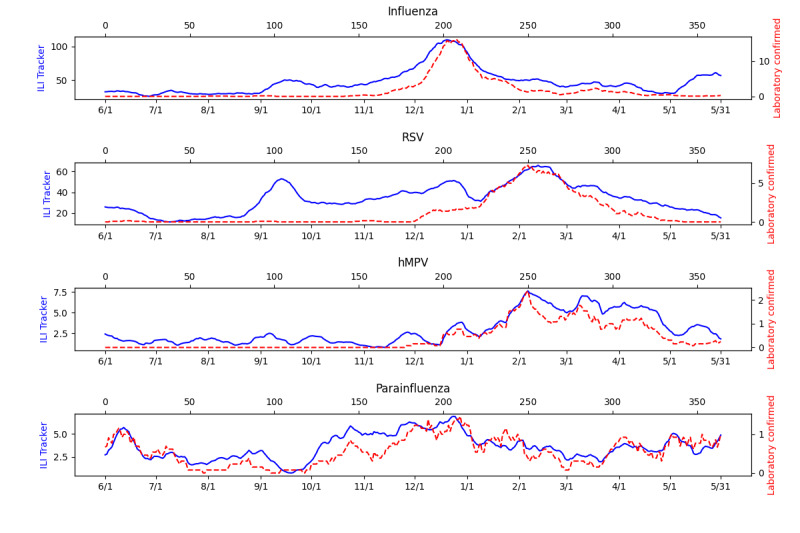
Expected and confirmed cases for June 1, 2014, to May 31, 2015 (7-day moving average). hMPV: human metapneumovirus; ILI: influenza-like illness; RSV: respiratory syncytial virus.

Figure 2 shows the results of running the ILI Tracker for the period June 1, 2014, to May 31, 2015. The red (dashed) lines are the daily number of laboratory-confirmed cases and the blue (solid) lines are the expected number of patients computed by ILI Tracker each day. The ILI-expected case predictions appear to be correlated with the number of positive laboratory results for those diseases. Note, however, that the expected and confirmed cases of RSV before January 1 deviate significantly. The possibility this deviation was due to a novel disease is discussed below. We computed the correlation between the daily number of expected and confirmed cases for each disease. Table 1 shows the Pearson and Spearman *r* and P values. Across the 4 modeled diseases, the peak days predicted by the ILI Tracker were close to the peak days according to the laboratory-confirmed cases. The peak days of the 7-day moving averages differed by 6 days for influenza, 6 days for RSV, 0 days for hMPV, and 4 days for PIV.

**Table 1:** Comparison of ILI Tracker and confirmed cases from June 1, 2014, to May 31, 2015, as measured using Pearson and Spearman correlations. The P values are the probability of the r values if the correlations were 0.

Disease	Pearson *r* (P value)	Spearman *r* (P value)
Influenza	0.81 (<.001)	0.63 (<.001)
RSV^a^	0.66 (<.001)	0.64 (<.001)
hMPV^b^	0.72 (<.001)	0.65 (<.001)
PIV^c^	0.51 (<.001)	0.52 (<.001)

^a^RSV: respiratory syncytial virus.

^b^hMPV: human metapneumovirus.

^c^PIV: parainfluenza.

### Putative Detection of a Novel Disease

The system reported finding a novel disease which, based on CDC reports for the time period being reported on, appears to be an enterovirus D68 (EV-D68) outbreak.

**Figure 3:**
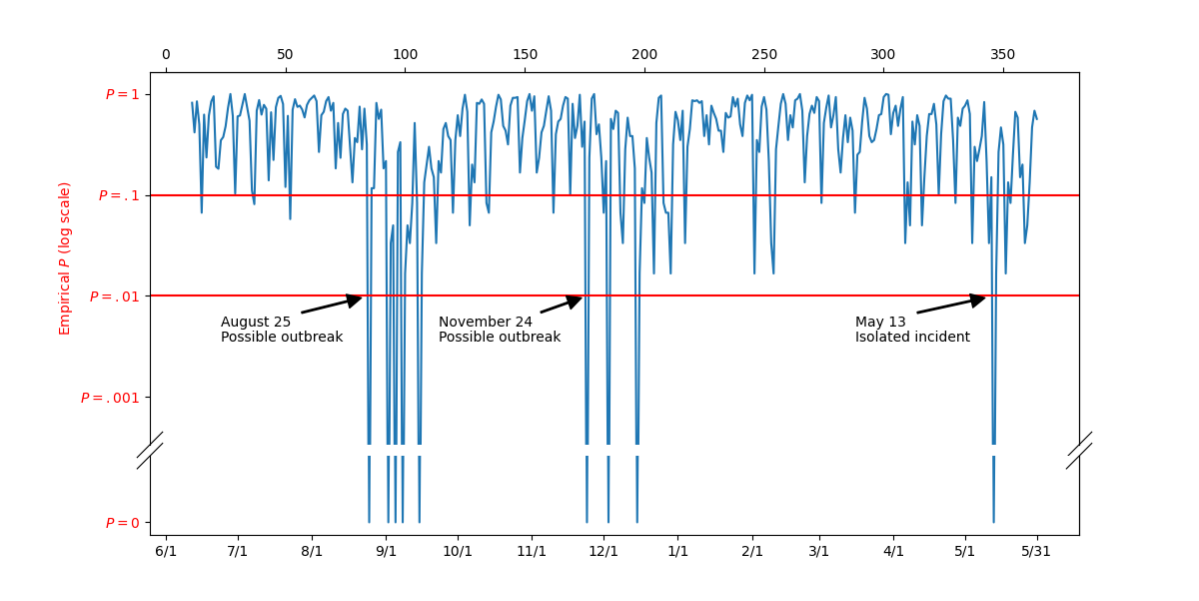
Daily empirical P values from June 1, 2014, to May 31, 2015.

Figure 3 shows the daily empirical P values from June 1, 2014, to May 31, 2015. The horizontal red lines indicate P=.1 and P=.01. As mentioned above, P=.01 is our threshold for unusual. On August 25, 2014, ILI Tracker signaled an unusual day. Although a single unusual day is not necessarily the beginning of an outbreak, it may warrant further investigation. A total of 8 of the 10 most unlikely patients on August 25 (ie, patients with very low probability findings given the expected prevalence of modeled diseases in the ED) showed signs of a respiratory illness. Starting on September 2, ILI Tracker noted 4 additional unusual days within a single week, with the most unlikely patients again showing signs of a respiratory illness. By September 9, there were sufficient data to characterize these anomalies in terms of the most prevalent findings.

Each day, *d*, we computed the expected number of patients with each finding, *f*, based on the rate of that finding for each disease and the prevalence of each disease in the ED on that day, with the formula 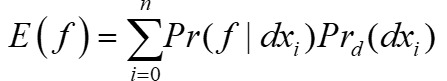.

**Figure 4:**
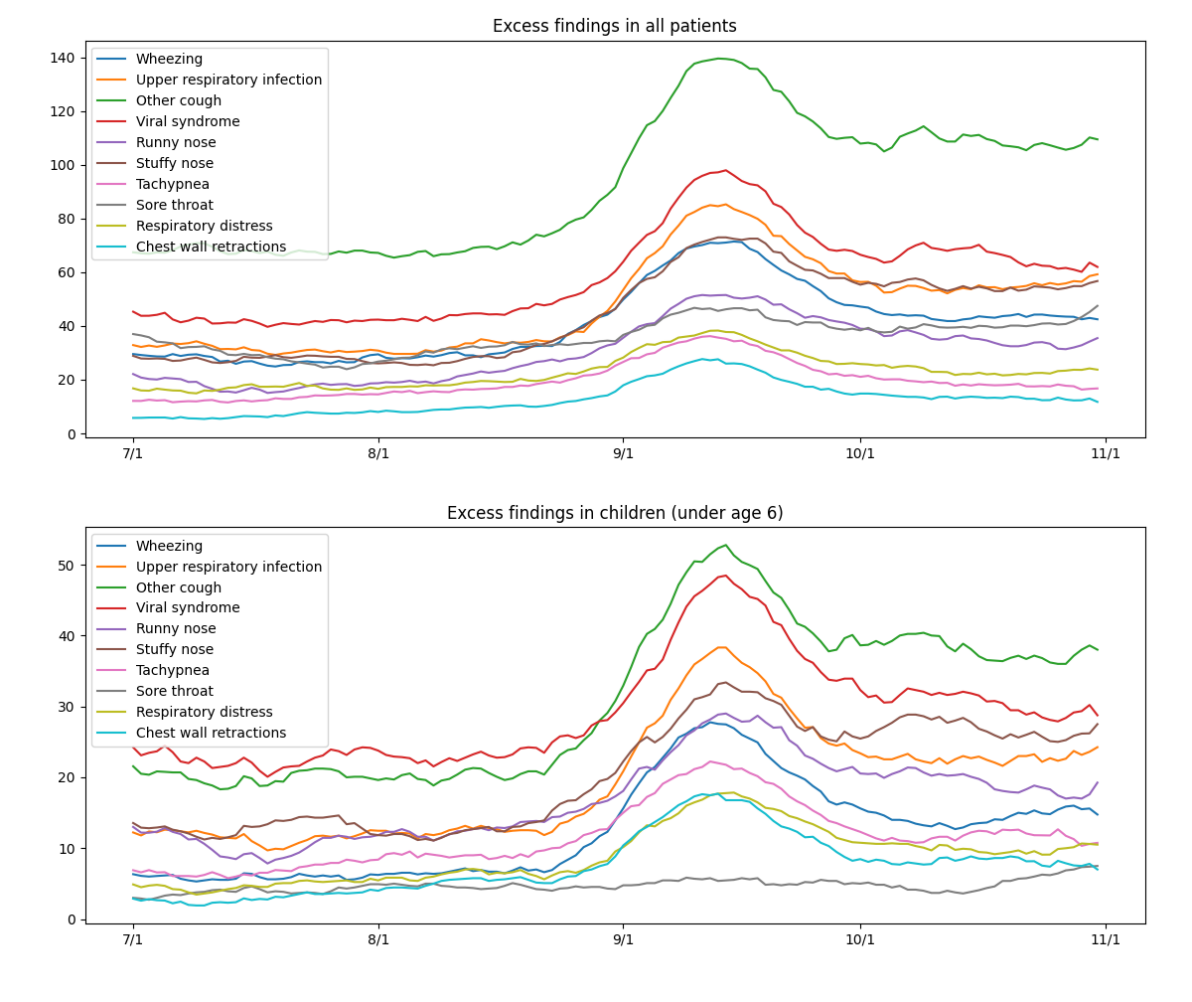
Daily absolute counts of the top 10 excess findings from July to October 2014.

If patients with a novel disease are present in the ED, we expect the actual number of findings in the data that are characteristic of the novel disease to exceed the expected number of those findings when assuming that only modeled diseases are present. We identified the top 10 most excessive findings for one week starting September 2. Figure 4 tracks the daily occurrence of these findings from July to October 2014. There was a clear increase in their frequency starting in the latter part of August.

During the late summer (late August and early September) of 2014, the ED of the UPMC Children’s Hospital of Pittsburgh experienced an abrupt increase in children presenting with acute respiratory illness, asthma exacerbation, and dyspnea. While symptoms overlapped with common causes of community-acquired viruses, they were unique based on the severity of illness, the sheer volume of children seeking care, and the timing being unusual for any of the annual common acute respiratory viruses that usually circulate. Rapid testing was negative for influenza and RSV. Even the full nucleic acid–based clinically available assays were confusing as some children tested positive for rhinovirus but were much more ill than expected. The assay was not supposed to cross-react with enterovirus, but it did.

During August and the fall of 2014, the CDC identified an outbreak of EV-D68 in the United States, especially among children [36]. As reported by the New Vaccine Surveillance Network (NVSN) [37], common symptoms of EV-D68 include cough, nasal congestion or rhinorrhea, wheezing, and shortness of breath or dyspnea [38]. These symptoms were among the top 10 excess symptoms identified by the ILI Tracker during the same time period (Figure 4). Although fever is often a common symptom of many ILIs, it was neither a common symptom identified by NVSN in children with EV-D68 nor was it among the excess symptoms identified by ILI Tracker. Neither Topaz nor the NVSN was designed to capture neurologic outcomes, such as acute flaccid myelitis, which is a rare but particularly severe neurologic consequence of EV-D68 leading to paralysis [39]. All these factors support that the novel disease identified by ILI Tracker is EV-D68.

On November 24, 2014, the ILI Tracker again signaled a day with highly unusual patient findings. This was a weaker signal (Figure 3) than the signal in late August and early September. Nine of the 10 most unusual patients showed signs of a respiratory illness. The ILI Tracker also noted an isolated unusual day on May 13, 2015.

### Experiment With a Synthetic Outbreak

**Figure 5:**
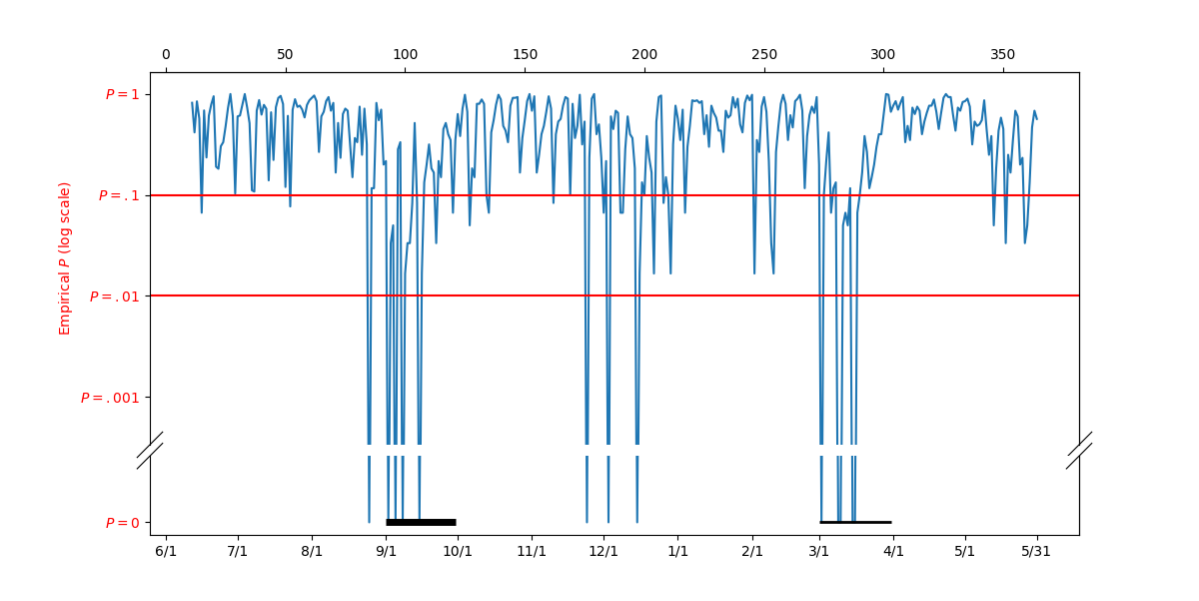
Daily empirical P values from June 1, 2014, to May 31, 2015, with a synthetic outbreak added to March 2015.

ILI Tracker identified a novel outbreak in late August and September of 2014. To further test the ILI Tracker, we created a synthetic outbreak by identifying cases from September 1, 2014, to September 30, 2014, with upper respiratory infection, respiratory distress, or chest wall retractions and artificially adding them from March 1 to 30, 2015. Figure 5 shows the empirical P values computed by ILI Tracker for the outbreak year June 1, 2014, to May 31, 2015, with this new artificial outbreak added to March. The thick black horizontal bars indicate where these cases were copied from (September 1-30) and the thin black horizontal bars indicate where they were copied to (March 1-30). A total of 2787 of these unusual cases from September 2014 were added to March 2015. The results in Figure 5 support that the ILI Tracker was able to identify this “outbreak” despite significant background activity from influenza, RSV, hMPV, and PIV during that time as shown in Figure 2. Note that the ILI Tracker produced a signal by the second day of March at which time only 152 of these cases had entered the ED. Note also that the ILI Tracker did not signal a novel outbreak in March when the cases of the artificial outbreak were not added to the data of that month.

### Comparison to an Alternate System

Autoregression is a standard method for outbreak detection [40-42]. To better understand the ability of the ILI Tracker to detect unmodeled outbreaks, we implemented an outbreak system based on autoregression and compared its performance with that of the ILI Tracker. We call the autoregression-based system AR Alarm.

For each finding, *f*, on each day, *d*, we built an AR(7) (autoregression with a lag term for each of the 7 previous days) model using the daily counts for that finding up to (but not including) the current day. We then used the model to predict the number of patients with that finding for the current day and called it *predicted(f,d).* We then set *p*(*f*,*d*)=*predicted*(*f*,*d*)/*pts*(*d*) where *pts*(*d*) is the total number of patients on day *d*. Thus, *p*(*f*,*d*) is the probability (predicted by the AR(7) model) that an arbitrary patient on day *d* has finding *f*. We then compute the probability (according to the AR(7) model) of seeing the actual number of patients with finding *f* on day *d, actual(f,d),* with the formula *Pr(actual(f,d))=Binom(actual(f,d),pts(d),p(f,d))*, where *Binom* is the binomial distribution. We use the Simple Bayes assumption and set the probability of the set of findings on the current day, *d*, to 
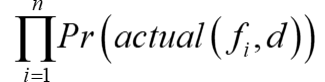
, where {*f_i_*} is the set of findings. We then compute an empirical P value of the findings on each day using the method described in the “Detecting the Presence of Unmodeled Diseases” section.

**Figure 6:**
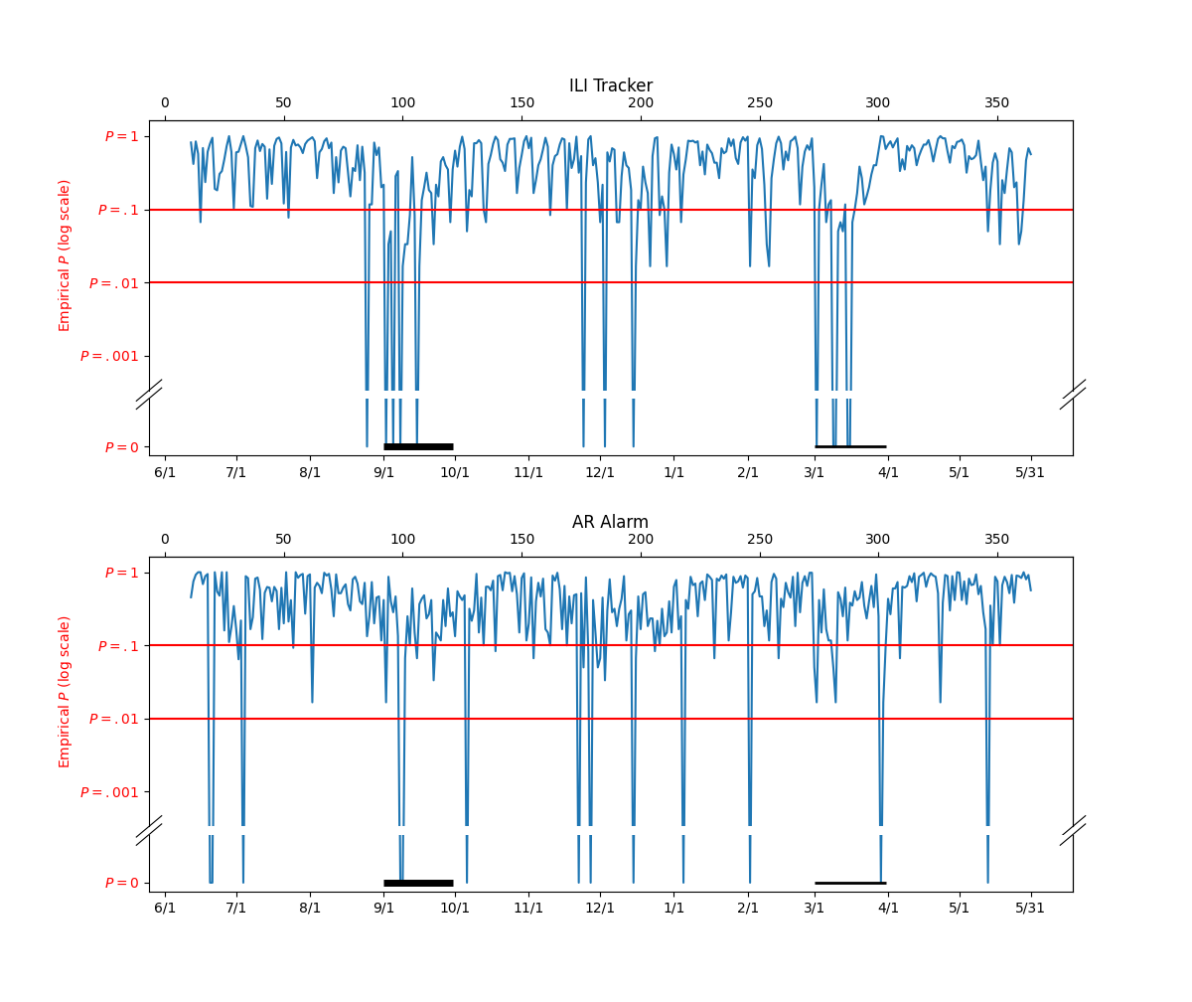
Comparison of empirical P values from ILI Tracker and AR Alarm for June 1, 2014 to May 31, 2015, with a synthetic outbreak added to March 2015. AR: autoregression; ILI: influenza-like illness.

Figure 6 compares the empirical P values for ILI Tracker to those from AR Alarm on the data from June 1, 2014, to May 31, 2015, including the artificial outbreak (described above) added to March*.* After the first 60 days, when the systems are calibrating themselves, they are in general agreement. Note, however, that ILI Tracker notices the August to September outbreak 14 days earlier than AR Alarm and with a stronger signal. Also, AR Alarm produces a few isolated signals, while ILI Tracker produces weaker signals (early October, early January, early February, and late March). Note that the ILI Tracker produces a strong signal and the AR Alarm produces a weaker signal in March when the artificial outbreak was added to the data*.*

### Runtime Analysis

Using a computer with 2 processors, each with six 1.6-GHz cores, it took less than 1 minute to construct all the disease models from the 4 years of training data.

Daily processing occurred in 3 phases. First, feature extraction from ED patient care reports. Second, computation of disease likelihoods for each patient. Third, computation of the expected number of each modeled disease and the P value of the data for each day.

On average, there were about 700 patients each day. Feature extraction typically took about 5 minutes each day using a computer with four 3.6-GHz cores. Computation of disease likelihoods typically took less than 1 minute for the entire set of patients on a given day. Given the likelihood for each patient, the ILI Tracker algorithm takes less than 10 seconds to compute the expected number of each modeled ILI and P value of the data each day using a computer with four 2.5-GHz cores. Thus, the total time to run the ILI Tracker per day on a desktop computer was less than 10 minutes.

We note that feature extraction and computation of disease likelihoods for each patient report are independent of the others. Thus, additional health care facilities can be added to our surveillance system and each can process their patient care reports using their own hardware, which would maintain run-time tractability. The runtime of the ILI Tracker algorithm for a given day is *O*(*P*×*D*×*F*) for each day, where *P* is the number of patients, *D* is the number of modeled diseases, and *F* is the number of modeled findings. Processing time increases linearly as patients, diseases, and features are added, and thus, the algorithm is readily scalable.

## Discussion

The performance of ILI Tracker during a 1-year period was moderate for tracking PIV, strong for tracking RSV and hMPV, and very strong for tracking influenza, as measured using Pearson correlation between the tracking and the laboratory-confirmed cases. Using Spearman correlation, ILI Tracker’s performance was moderate for tracking PIV and strong for tracking hMPV, RSV, and influenza.

As mentioned above, in late August 2014, ILI Tracker alerted on an outbreak consistent with the EV-D68 outbreak that was identified as present in the United States during that period by the CDC. To our knowledge, that EV-D68 outbreak is the only novel outbreak that was documented to have occurred during the period of our study (June 1, 2014, to May 31, 2015). ILI Tracker is intended to be used as a daily monitor that has its alerts interpreted by clinicians and public health officials. It can identify and output unusual patients for further evaluation by such individuals. Approaches that use aggregate statistics (based on overall counts of findings) cannot identify individual patients who are likely to have an outbreak disease.

If ILI Tracker had been in operation during 2014 it would have signaled a statistical anomaly among patients in late August and provided a set of patients for further investigation*.* Based on clinical judgment, these patients could be assessed, tested, and possibly isolated. In some cases, samples might be obtained for rapid sequencing. By early September, ILI Tracker could also have provided a preliminary clinical description and timeline of a cohort of unusual patients who turned out to have findings consistent with an outbreak of EV-D68. In the future, such information could help clinicians and public health officials to detect, isolate, characterize, and identify such a novel disease early in the outbreak.

A putative outbreak of an unmodeled disease also appears to have occurred in late November and December of 2014 during outbreaks of both RSV and influenza. Although these patients could easily be lost among a large number of patients with RSV and influenza, they are statistically unlikely to be among those known diseases, according to the analysis by ILI Tracker. Although this was a weak signal, it was statistically significant enough to warrant further investigation. Again, if ILI Tracker had been in operation at that time, it would have identified patients for further evaluation. The source of that putative outbreak remains an open question.

The unusual day detected on May 13, 2015 is likely an isolated incident. Statistically, we should expect such incidents to occur occasionally. ILI Tracker would provide a set of candidate patients for consideration by clinicians and public health officials in the region.

The results reported in this paper provide support that ILI Tracker was able to track well 4 modeled ILI-like diseases over a 1-year period, relative to laboratory-confirmed cases, and it was computationally efficient in doing so. The results we presented also provide support that the system was able to detect a novel outbreak of EV-D68 early in an outbreak that occurred in Allegheny County in 2014, as well as clinically characterize that outbreak disease accurately. Detection was very efficient computationally. In general, the ILI Tracker scales linearly in the number of diseases and number of findings per disease. Thus, it can be expanded to model many additional, known outbreak diseases and their findings.

This work has some important limitations that we plan to address in the future. Here, we assumed a small set of possible (unique) diagnoses. In future work, we plan to extend ILI Tracker to model additional (potentially co-occurring) respiratory diseases, including adenovirus, enterovirus, and SARS-CoV-2. We currently use the Topaz parser to extract a set of less than 100 findings selected specifically for their relevance to ILIs. We plan to use the MetaMap system [43] to expand the set of findings to many thousands that are encoded using Unified Medical Language System concept unique identifiers [44]. In addition, we plan to evaluate ILI Tracker and its extensions on additional years of data from a broader range of hospitals. We implicitly assume that patient care does not vary over time, reporting is constant and comprehensive, and mild cases are noted and documented (regardless of their primary diagnosis).

Our ultimate goal is to deploy an effective, free, and open-source early-warning surveillance system for use in monitoring data in hospital EDs. A preliminary version of the ILI Tracker software will be available on GitHub [45] with updates and test data planned for the future.

## Abbreviations

AR(7): autoregression with a lag term for each of the 7 previous days
AUC: area under the receiver operating characteristic curve
CDC: Centers for Disease Control and Prevention
ED: emergency department
EV-D68: enterovirus D68
hMPV: human metapneumovirus
ILI: influenza-like illness
NVSN: New Vaccine Surveillance Network
PIV: parainfluenza
RSV: respiratory syncytial virus
UPMC: University of Pittsburgh Medical Center
